# Supplementary material for: Some structural features of the peptide profile of myelin basic protein-hydrolyzing antibodies in schizophrenic patients
Source: PeerJ. 2023 Jul 6;11:e15584. doi: 10.7717/peerj.15584 (PMC10329820; doi:10.7717/peerj.15584)
Supplement: Figure S1 — Each data point indicates the correspondence of measured integral MS signal of peptide ion (XIC value) in IgG sample ( x-axis) and measured proteolytic activity of this IgG ( y-axis). Labels correspond to m/z of peptide ion shown in legend. [file peerj-11-15584-s003.pdf]

**Figure S1.** Dependence of the abundance of selected features on proteolytic activity of IgG against MBP.

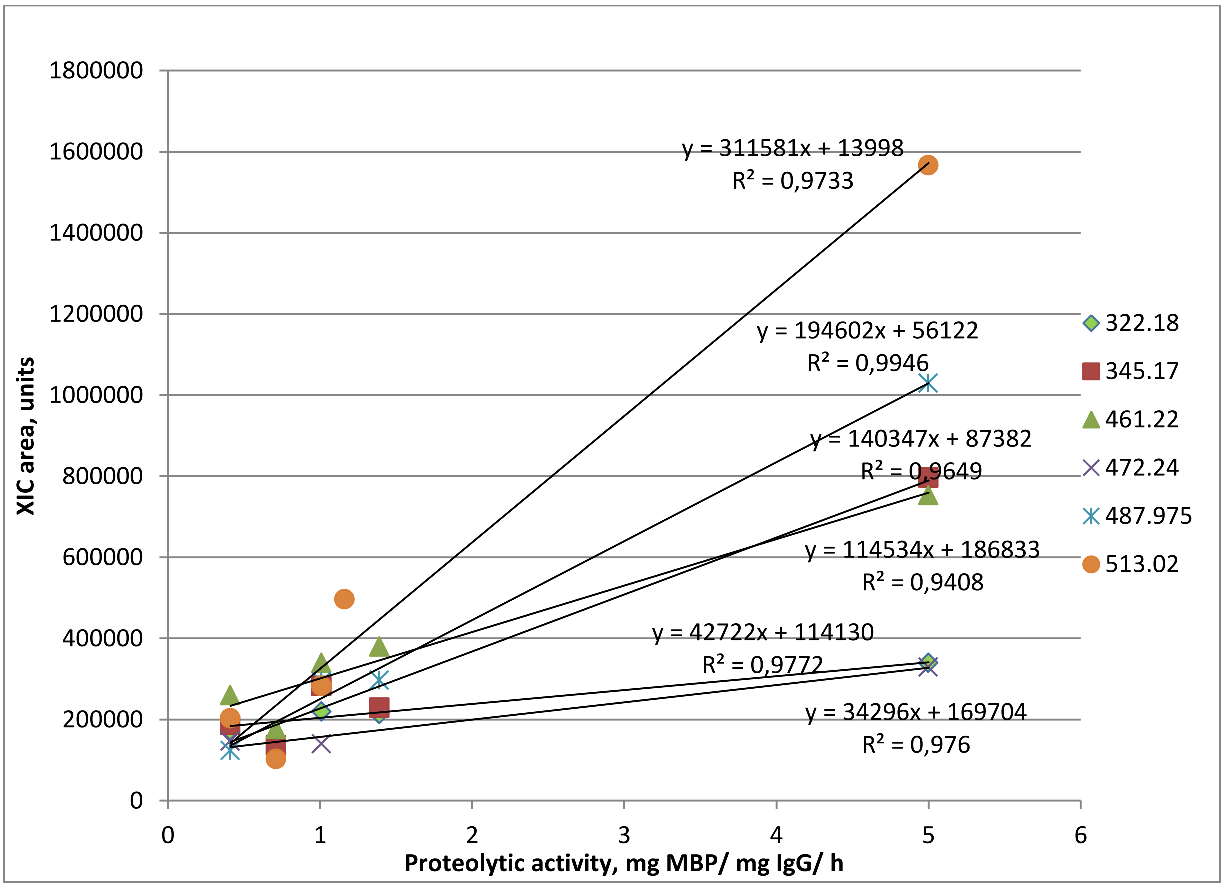


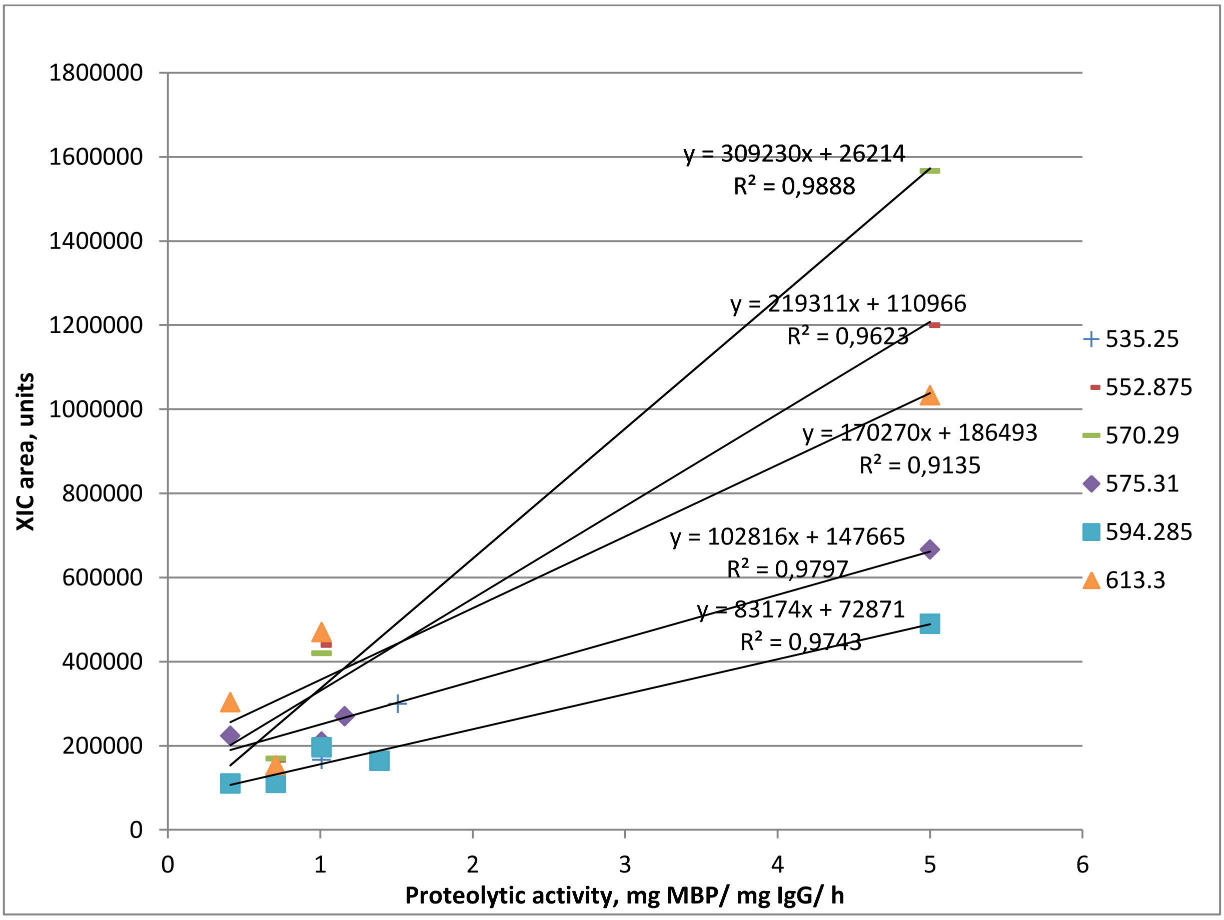

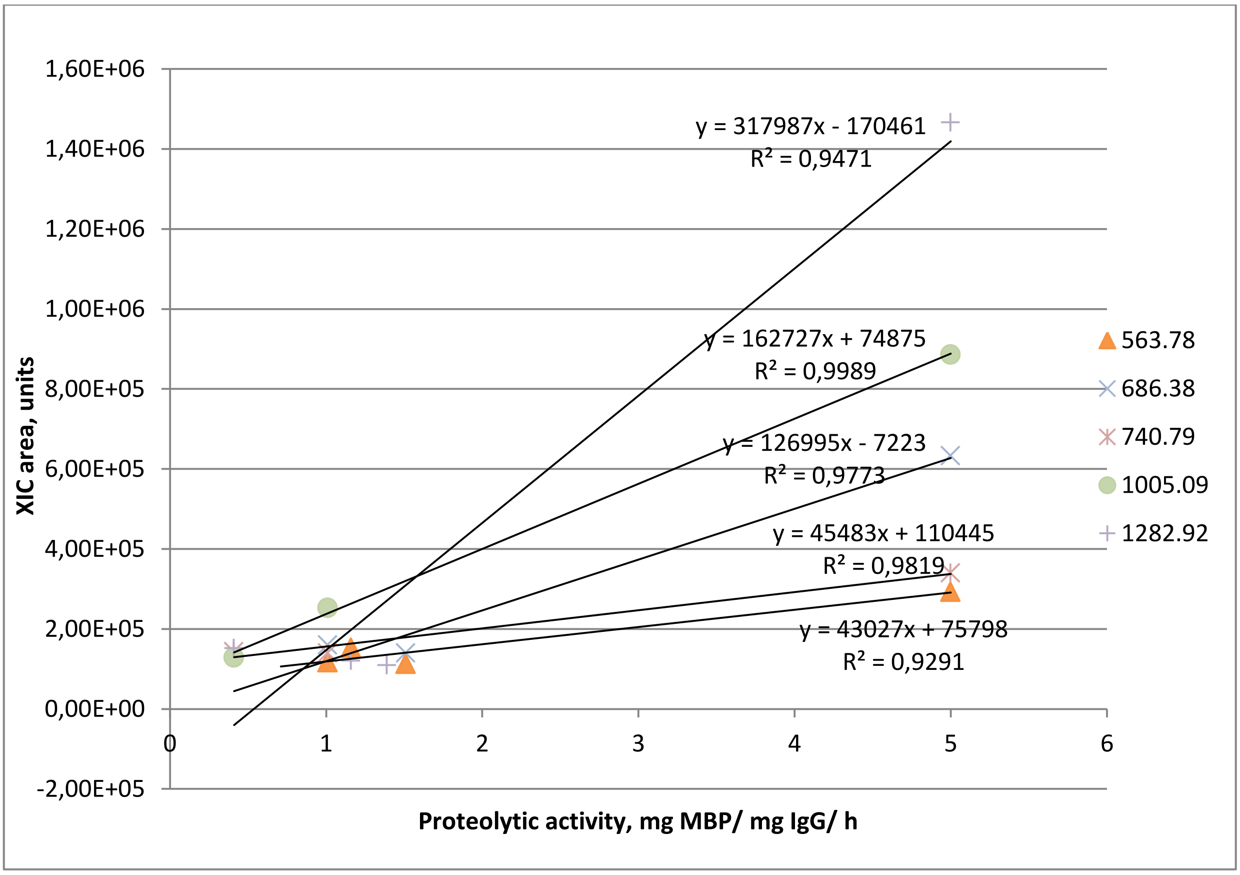

1,60E+06
1,40E+06
1,20E+06
1,00E+06
8,00E+05
6,00E+05
4,00E+05
2,00E+05
0,00E+00
-2,00E+05
0
1
2
3
4
5
6
XIC area, units
Proteolytic activity, mg MBP/ mg IgG/ h
y = 317987x - 170461
R² = 0,9471
y = 162727x + 74875
R² = 0,9989
y = 126995x - 7223
R² = 0,9773
y = 45483x + 110445
R² = 0,9819
y = 43027x + 75798
R² = 0,9291
563.78
686.38
740.79
1005.09
1282.92
