# Supplementary material for: Some structural features of the peptide profile of myelin basic protein-hydrolyzing antibodies in schizophrenic patients
Source: PeerJ. 2023 Jul 6;11:e15584. doi: 10.7717/peerj.15584 (PMC10329820; doi:10.7717/peerj.15584)
Supplement: Figure S2 [file peerj-11-15584-s004.pdf]

Correspondence of b- and y-fragments of the predicted peptide sequences to MS/MS spectra of selected m/z features.

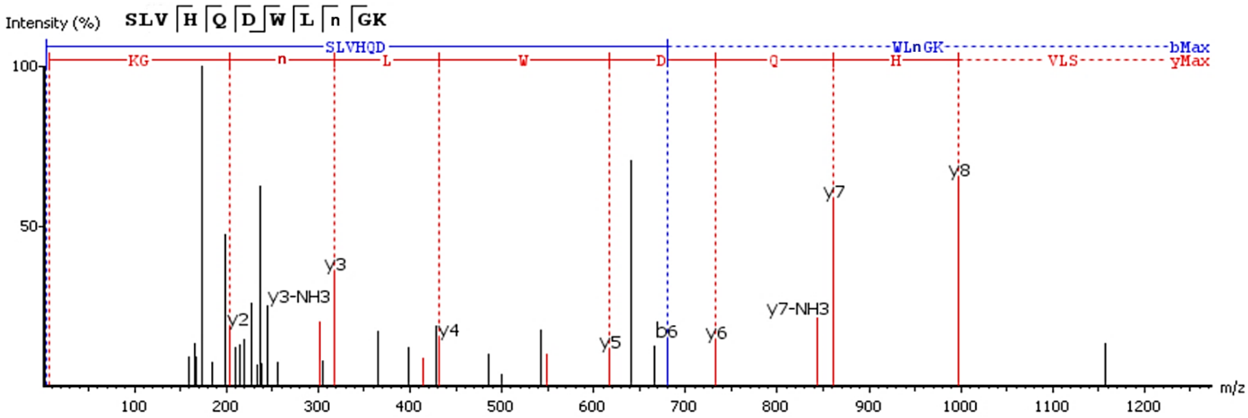

Figure S1.1. Correspondence of b- and y-fragments of the predicted peptide SLVHQDWLN(–17)GK to MS/MS spectrum of m/z = 427.219^{3+}. Peptide sequence has been corrected to SVLHQDWLN(–17)GK after BLAST alignment and validation by Peptide fragmentation modeling utility.

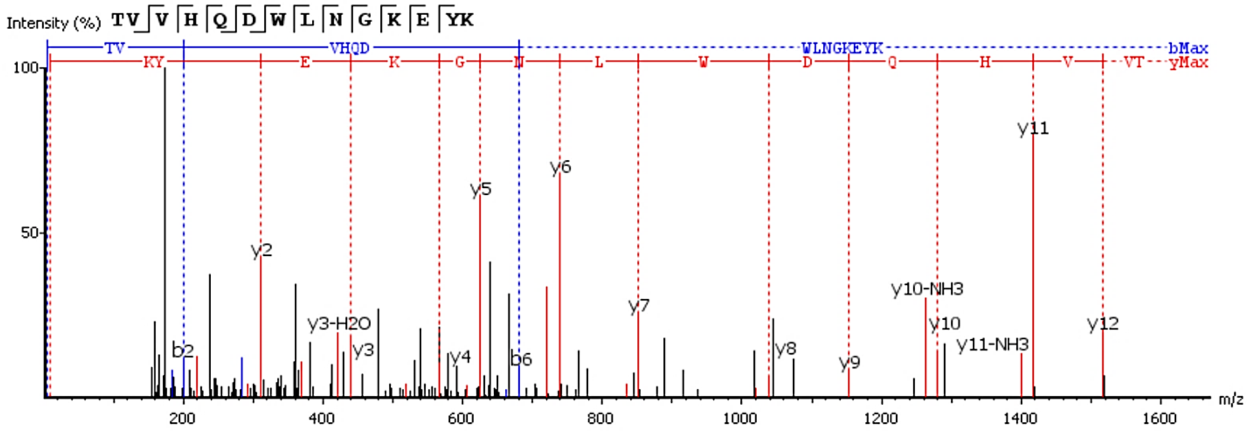

Figure S1.2. Correspondence of b- and y-fragments of the predicted peptide TVVHQDWLN(+0.98)GKEYK or TVVHQN(+0.98)WLDGKEYK to MS/MS spectrum of m/z = 430.219^{4+}.

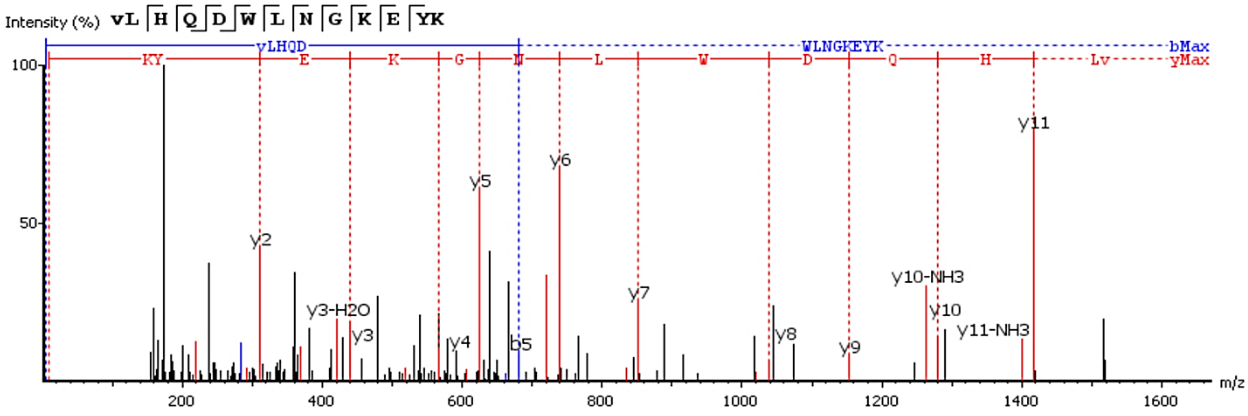

Figure S1.3. Correspondence of b- and y-fragments of the predicted peptide SVLHQDWLN(+0.98)GKEYK or SVLHQN(+0.98)WLDGKEYK to MS/MS spectrum of m/z = 430.219^{4+}.

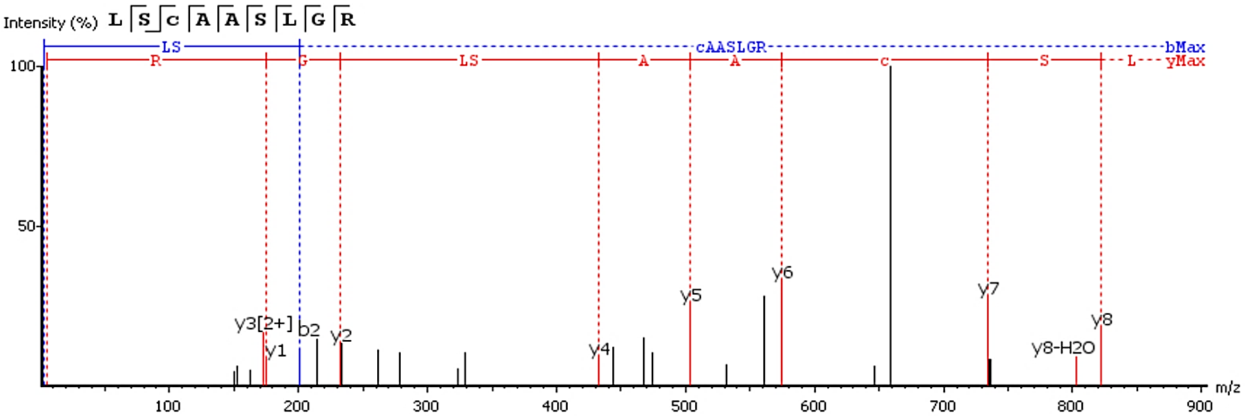

Figure S1.4. Correspondence of b- and y-fragments of the predicted peptide LSC(+57)AASGLR to MS/MS spectrum of m/z = 467.742^{2+}.

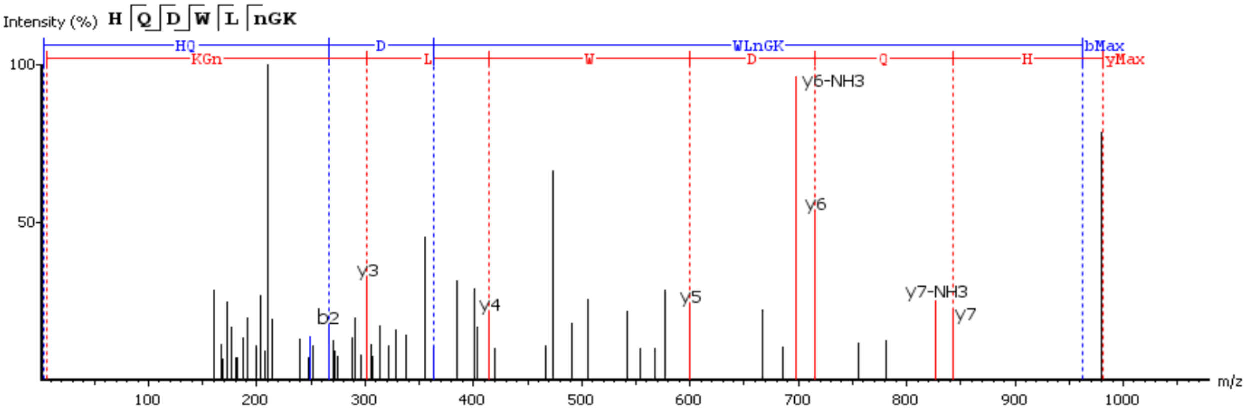

Figure S1.5. Correspondence of b- and y-fragments of the predicted peptide HQDWLN(–17)GK to MS/MS spectrum of m/z = 490.733^{2+}.

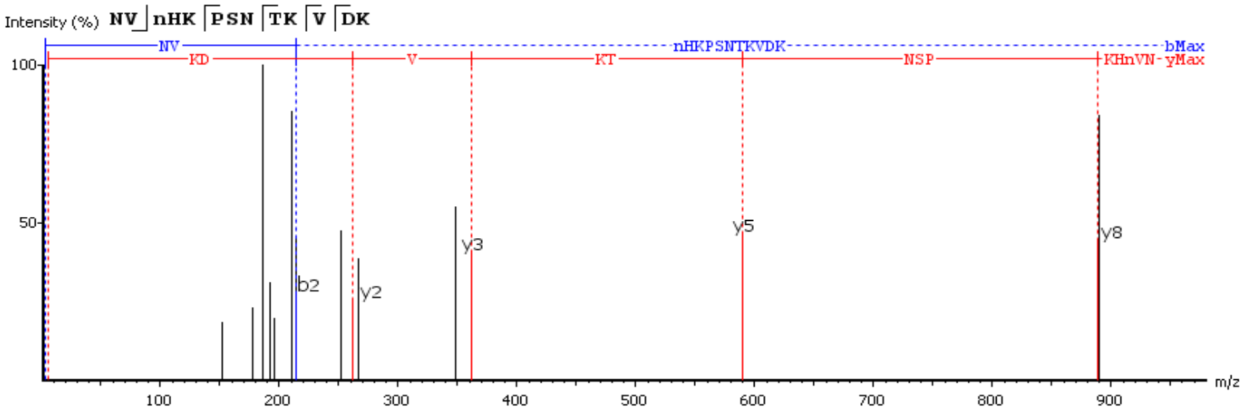

Figure S1.6. Correspondence of b- and y-fragments of the predicted peptide NVN(+0.98)HKPSNTKVDK to MS/MS spectrum of m/z = 494.596^{3+}.

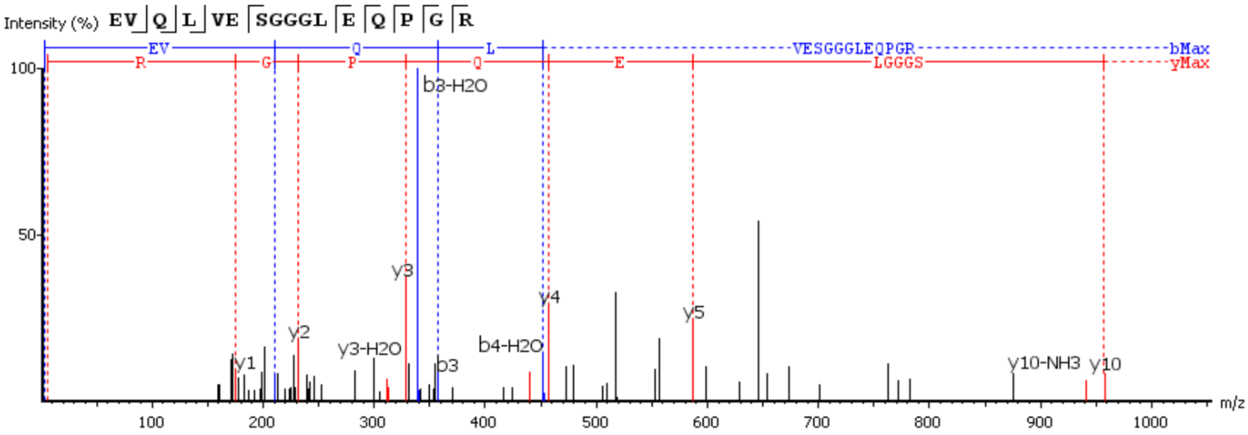

Figure S1.7. Correspondence of b- and y-fragments of the predicted peptide EVQLVESGGGLEQPGR to MS/MS spectrum of m/z = 552.29^{3+}.

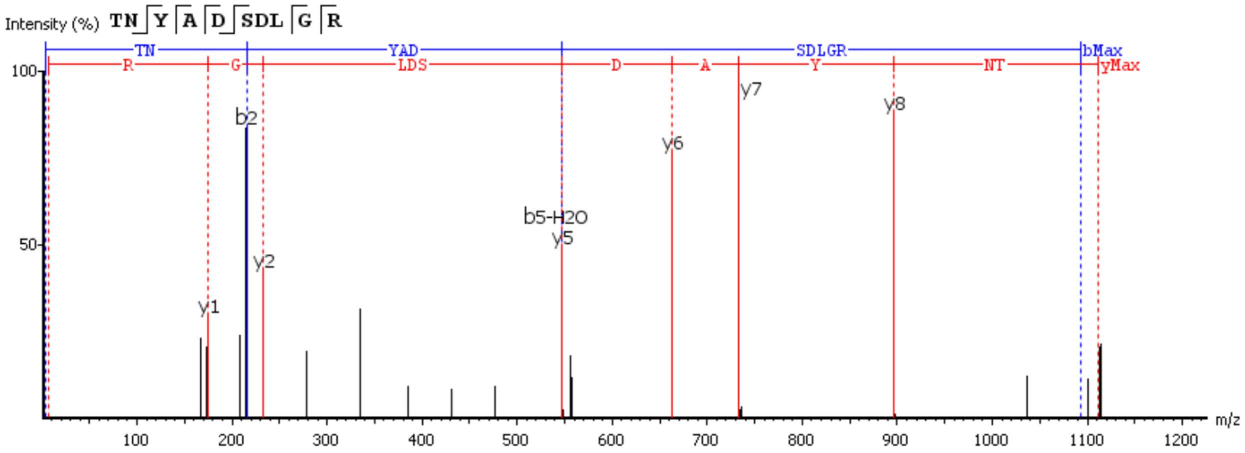

Figure S1.8. Correspondence of b- and y-fragments of the predicted peptide TNYADSDLGR to MS/MS spectrum of m/z = 556.254^{2+}. Di-peptides DL and VE are equivalent in mass, and peptide sequence has been corrected to TNYADSVEGR after BLAST alignment.

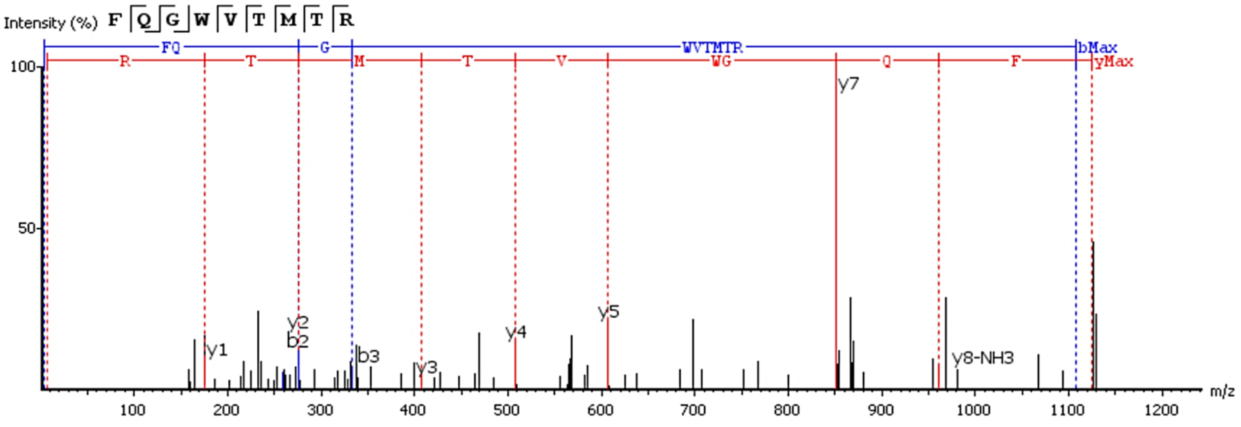

Figure S1.9. Correspondence of b- and y-fragments of the predicted peptide FQ(+0.98)GWVTMTR to MS/MS spectrum of m/z = 563.78^{2+}.

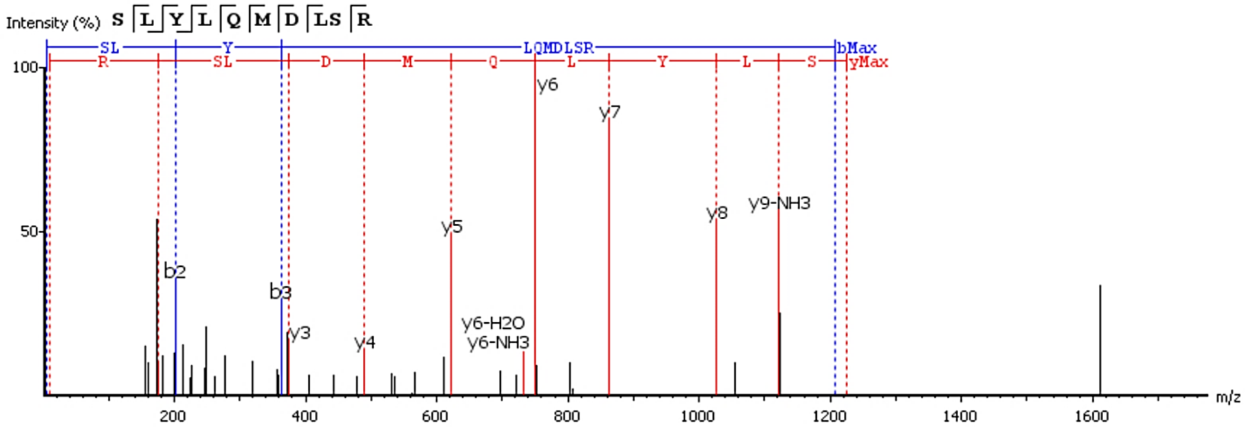

Figure S1.10. Correspondence of b- and y-fragments of the predicted peptide *LYLQMN(+0.98)SLR to MS/MS spectrum of m/z = 613.3^{3+}.

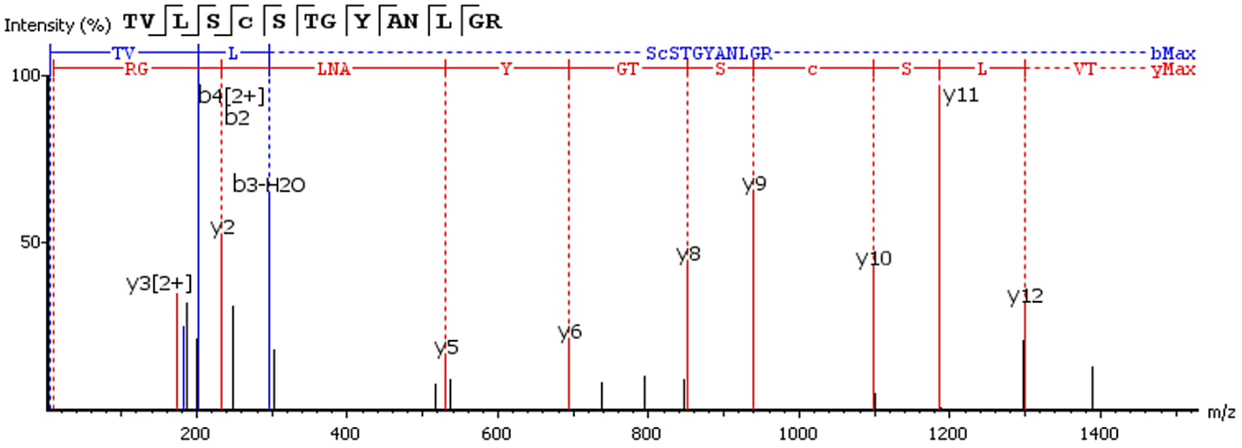

Figure S1.11. Correspondence of b- and y-fragments of the predicted peptide VTLSC(+57)SGTYANLGR to MS/MS spectrum of m/z = 749.869^{2+}.

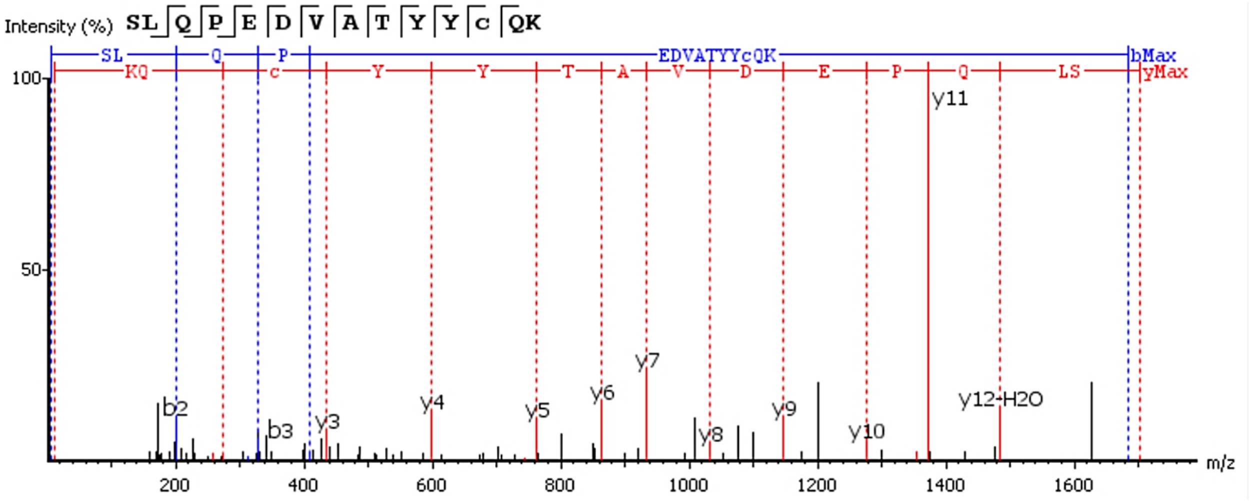

Figure S1.12. Correspondence of b- and y-fragments of the predicted peptide SLQPEDVATYYC(+57)QK to MS/MS spectrum of m/z = 851.393^{2+}.
