# Supplementary figures and images for: Some structural features of the peptide profile of myelin basic protein-hydrolyzing antibodies in schizophrenic patients
Source: PeerJ. 2023 Jul 6;11:e15584. doi: 10.7717/peerj.15584 (PMC10329820; doi:10.7717/peerj.15584)

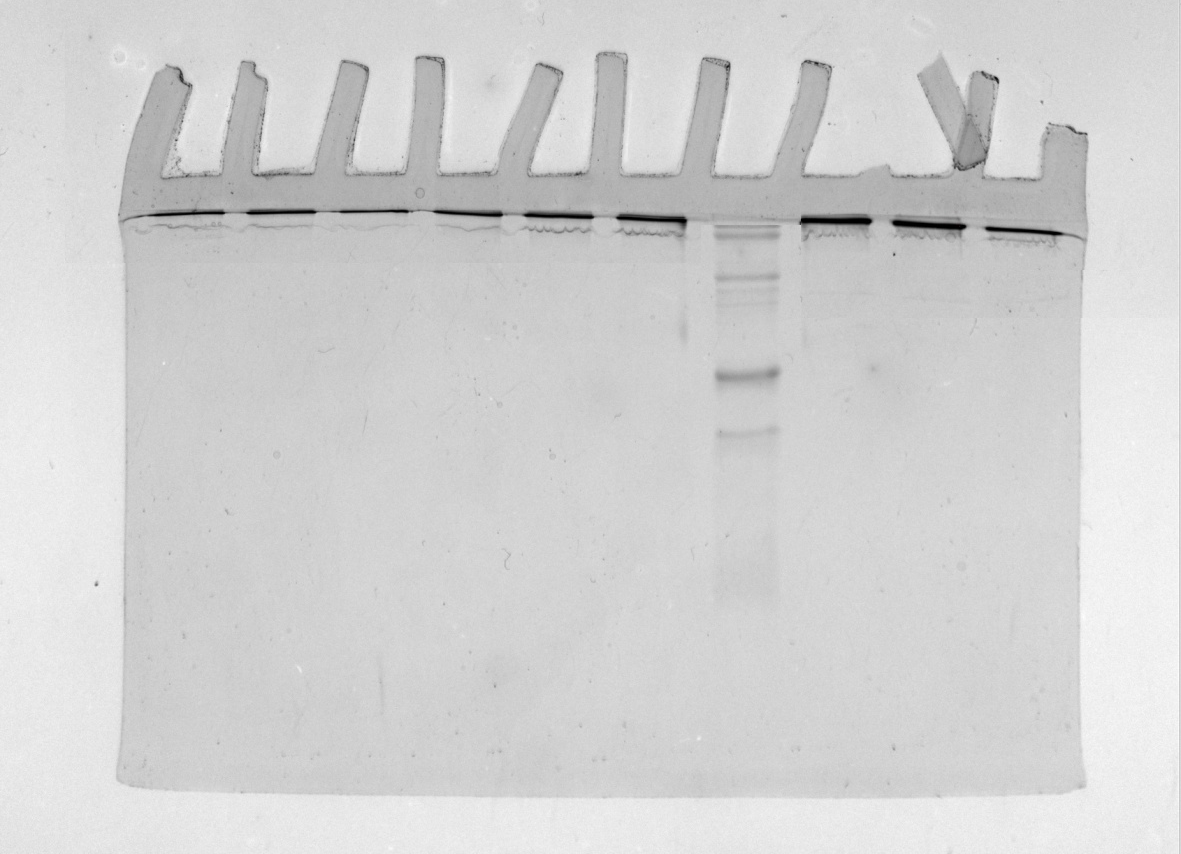

Supplement: Supplemental Information 5 [file peerj-11-15584-s005.zip › Figure 1 uncropped.tiff]
